# Correction: Development and application of multiplex targeted-sequencing approaches to identify *Phytophthora* species associated with root rot and wilting complex of red raspberry

**DOI:** 10.1371/journal.pone.0308578

**Published:** 2024-08-05

**Authors:** The PLOS ONE Editors

## Notice of Republication

An incorrect copyright license was mistakenly applied to this article [1] upon publication. The publisher apologizes for the error. At the time of publication of this notice, the article [1] was republished to update the Copyright license and statement. Please download this article again to view the correct version.

## References

[1] Sapkota S, Burlakoti RR, Lamour K, Lubberts M, Punja ZK (2022) Development and application of multiplex targeted-sequencing approaches to identify *Phytophthora* species associated with root rot and wilting complex of red raspberry. PLoS ONE 17(11): e0275384. doi: 10.1371/journal.pone.0275384 36417394 PMC9683591

