# Correction: Correction: Development and application of multiplex targeted-sequencing approaches to identify *Phytophthora* species associated with root rot and wilting complex of red raspberry

**DOI:** 10.1371/journal.pone.0309249

**Published:** 2024-08-15

**Authors:** The PLOS ONE Editors

The *PLOS ONE* Editors issue this notice to correct an error in the correction notice [1] for this article [2]. Contrary to the statement in the original correction notice [1], the article [2] was not republished at the time of the publication of the notice, but instead was republished on August 9, 2024. The Copyright license and statement of the article [2] have now been updated. Please download this article again to view the correct version.

The publisher apologizes for the error.

## References

[1] The *PLOS ONE* Editors (2024) Correction: Development and application of multiplex targeted-sequencing approaches to identify *Phytophthora* species associated with root rot and wilting complex of red raspberry. PLoS ONE 19(8): e0308578. 10.1371/journal.pone.0308578 39102393 PMC11299800

[2] Sapkota S, Burlakoti RR, Lamour K, Lubberts M, Punja ZK (2022) Development and application of multiplex targeted-sequencing approaches to identify *Phytophthora* species associated with root rot and wilting complex of red raspberry. PLoS ONE 17(11): e0275384. 10.1371/journal.pone.0275384 36417394 PMC9683591

